# Does Male Circumcision Protect against Sexually Transmitted Infections? Arguments and Meta-Analyses to the Contrary Fail to Withstand Scrutiny

**DOI:** 10.1155/2014/684706

**Published:** 2014-05-13

**Authors:** Brian J. Morris, Catherine A. Hankins, Aaron A. R. Tobian, John N. Krieger, Jeffrey D. Klausner

**Affiliations:** ^1^School of Medical Sciences and Bosch Institute, University of Sydney, Sydney, NSW 2006, Australia; ^2^Department of Global Health, Academic Medical Centre and Amsterdam Institute for Global Health and Development, University of Amsterdam, 1100DE, Amsterdam, The Netherlands; ^3^Department of Infectious Disease Epidemiology, Faculty of Epidemiology and Population Health, London School of Hygiene and Tropical Medicine, London, WC1E 7HT, UK; ^4^Department of Pathology, School of Medicine, Johns Hopkins University, Baltimore, MD 21287, USA; ^5^Section of Urology University of Washington School of Medicine and VA Puget Sound Health Care System, Seattle, WA 98108, USA; ^6^Division of Infectious Diseases and Program in Global Health, David Geffen School of Medicine, University of California Los Angeles (UCLA), Los Angeles, CA 90095, USA

## Abstract

We critically evaluate a recent article by Van Howe involving 12 meta-analyses that concludes, contrary to current evidence, that male circumcision increases the risk of various common sexually transmitted infections (STIs). Our detailed scrutiny reveals that these meta-analyses (1) failed to include results of all relevant studies, especially data from randomized controlled trials, (2) introduced bias through use of inappropriate control groups, (3) altered original data, in the case of human papillomavirus (HPV), by questionable adjustments for “sampling bias,” (4) failed to control for confounders through use of crude odds ratios, and (5) used unnecessarily complicated methods without adequate explanation, so impeding replication by others. Interventions that can reduce the prevalence of STIs are important to international health. Of major concern is the global epidemic of oncogenic types of HPV that contribute to the burden of genital cancers. Meta-analyses, when well conducted, can better inform public health policy and medical practice, but when seriously flawed can have detrimental consequences. Our critical evaluation leads us to reject the findings and conclusions of Van Howe on multiple grounds. Our timely analysis thus reaffirms the medical evidence supporting male circumcision as a desirable intervention for STI prevention.

## 1. Introduction

Male circumcision has long been considered to have a protective effect against acquisition of various sexually transmitted infections (STIs) [1–5]. This benefit is one of many that have led to recent affirmative evidence-based policy statements by medical bodies in support of the procedure [6, 7]. At the same time there is vigorous opposition to the procedure by fringe groups whose campaigning is based on a diversity of arguments [8]. To date these have been consistently exposed as fallacious (see, e.g., [9] and the references therein).

The issue of male circumcision for protection against STIs is one of the potential benefits that opponents dispute, sometimes with the support of data. Their arguments have been fully enunciated in a recent 42-page article that included a series of 12 meta-analyses [10]. We therefore decided to make that article the focus of our paper. Its author, Van Howe, claimed that his analyses show that male circumcision actually* increases* STI risk. His marathon study examined STIs other than human immunodeficiency virus (HIV),* Trichomonas vaginalis,* and* Mycoplasma genitalium.* It concluded,* “Most specific STIs are not impacted significantly by circumcision status. These include chlamydia, gonorrhea, HSV, and HPV” *and that,* “Consequently, the prevention of STIs cannot be rationally interpreted as a benefit of circumcision, and a policy of circumcision for the general population to prevent STIs is not supported by the evidence currently available in the medical literature” *[10]. His study has, at first glance, upped the ante in this debate by calling into question the current medical position.

When performed well, meta-analysis, by combining data arising from different studies, can substantially increase the “*n*” value and thus the power to see an effect and in the process smooth over discrepancies between studies so as to reach a more reliable conclusion than would not otherwise have been possible if single, sometimes disparate, data were considered alone. High quality meta-analyses and large, well-designed randomized controlled trails (RCTs) are each regarded as level 1++ evidence, the highest rating ascribed to any study [11]. They therefore have the potential to form a firm basis for medical decision making and health policy formulation.

Given the important implications of Van Howe's extensive, single author article, we considered it imperative that it be subjected to a careful, detailed evaluation by experts in the scientific community before his position and that of other opponents become accepted by the wider medical profession and health authorities. Our aim was thus to provide the necessary “forensic” evaluation of the opposing side of the debate.

We first examine in detail the particular statistical methods adopted and whether there are any drawbacks to these. For each STI we then examine the search strategy used, whether all relevant references, especially RCTs, were included and used in each meta-analysis, whether manipulations of unadjusted results were appropriate, the validity of adjustments for* “lead-time bias,” “sampling bias,”* and* “publication bias,”* and whether manipulation of control groups was appropriate. This is followed by an appraisal of reasoning and results from the meta-analyses presented of male circumcision and each STI studied, namely, human papillomavirus (HPV), genital warts, nongonococcal (nonspecific) urethritis (NGU),* Chlamydia trachomatis*,* Neisseria gonorrhoea*, genital herpes/*Herpes simplex *virus (HSV) type 2, genital discharge syndrome (GDS), genital ulcerative disease (GUD), syphilis, chancroid, and STIs in general. Finally we examine other issues raised in support of the contrary argument in this debate, before arriving at an appropriate evidence-based conclusion.

Since (i) the conclusions of opponents and this recent article in particular are sufficiently at odds with the understanding most researchers have of the literature on STIs and male circumcision and that (ii) it is well known that statistics can be used for obfuscation, our critical appraisal should help provide assurance to workers in the field puzzled by the contrary findings.

## 2. General Methodological Considerations

### 2.1. Statistical Methods Adopted for Meta-Analyses

The statistical techniques employed by Van Howe are considerably more complicated than can be found in a standard meta-analysis. They are sufficiently advanced that casual readers, and indeed many researchers in the field would be unable to ascertain whether the statistical methods are appropriate or not. A more detailed discussion of the basis of these is provided in the next section. Their adoption could have been in response to criticism [12] of the author's initial foray into meta-analyses in 1999 (on male circumcision and HIV) [13], in which the mistake of simple data pooling [14] led to a Simpson's paradox (also known as the Yule-Simpson effect, the “reversal paradox,” or the “amalgamation paradox”) [15]. This paradox is when a trend that appears in different groups of data disappears when these groups are combined, and the reverse trend appears for the aggregate data. The problem is often encountered in social science and medical science statistics [16] and is particularly confounding when frequency data are unduly given causal interpretations [17]. A textbook on meta-analyses [18] and a review of methods and techniques in meta-analyses [19] use this meta-analysis as an illustrative example of how Simpson's paradox can lead to incorrect results. Subsequent, correctly performed, meta-analyses found male circumcision to have a strong protective effect against HIV infection [20, 21].

### 2.2. Use of Crude Odds Ratios

The article uses the term* “exact odds ratio”* for the data drawn from each study in the article's Tables 3–14. A more accurate term would be “exact crude odds ratio,” “crude” because the odds ratios (ORs) are not adjusted for other factors, and “exact” because of the statistical method used to calculate the OR and confidence intervals (CIs). “Exact” ORs are a complicated area of statistics.

We will first explain the different types of OR. An OR is the ratio between two pairs of odds. Each of the odds obeys a binomial distribution. For simplicity in calculations (especially for the CIs) an assumption is made that the data follow the normal (Gaussian) distribution, since this makes calculation of the OR and CI straightforward. While not exact, when *n* values are sufficiently large, the distribution of the data tends to follow a normal distribution. However, this assumption of a normal distribution becomes notably less true when *n* values are very small, for example, when any of the frequencies are ≤5 or the total *n* value is ≤20. In contrast, “exact” ORs (and “exact” CIs) are calculated using methods that work directly with the binomial distribution. These “exact” OR calculations are quite complex, requiring expensive software and very fast computers that can require an hour or more to calculate each result. Derivation of such exact ORs in meta-analyses is unusual and generally considered unnecessary. Van Howe's argument is that studies sometimes match the conditions when normal approximation is invalid, and therefore it is better to use exact methods. While this point may be true, when a study does meet such conditions it is likely to have plenty of variance, meaning that it will not have much weight in the meta-analysis anyway. Tables 3–14 in his article include a column showing use of an* “exact odds ratio” *for each original study included as source data for each meta-analysis. He did this rather than using normal approximations, in spite of the fact that these calculations rarely make much difference in a meta-analysis. Generally speaking the only time normal approximations are substantially different from approximate ORs is when the CIs are so wide that they would have limited influence on the summary ORs anyway.

A further concern is the use of exact ORs as input to the meta-analysis. Because meta-analysis assumes a normal approximation anyway, the approach adopted loses some of the “exactness” that so much effort was put into achieving. So the endeavors in going to all of the trouble to calculate exact ORs can be regarded as an exercise in futility given that meta-analysis is an approximate method. While neither of these concerns are arguments against using exact ORs, we do wonder how much value they offer. More importantly, whereas it does not do any harm as such, using “exact” ORs makes it difficult for those who lack the sophisticated software and computer power to verify the results obtained. This is a good reason for not using such methods.

Apart from the issue of whether crude OR are “exact” or not, a more important issue is that the article regards use of exact crude ORs as a strength. In fact it is a weakness. The reasons were explained previously in a critique by Waskett et al. [22] of a 2007 meta-analysis on sexually transmitted urethritis, where those critics stated,* “Another error is that Van Howe appears to present crude measures of association between circumcision and sexually transmitted infection (STI), even though adjusted figures are available for many studies, and are more appropriate because they partially control for confounding, such as by religion or sexual behaviour. For example, Diseker et al.* (our reference[23])* reported adjusted *ORs* of 1.3 and 1.6 for the association of gonorrhoea and lack of circumcision in cross-sectional and cohort analyses, respectively, but Van Howe cites the crude *ORs* of 1.09 and 1.24.”*.

Van Howe's article justifies calculation of exact crude ORs from frequency data arguing that regression models can be manipulated to produce a desired result. The problem is that observational studies in particular are highly susceptible to confounding factors. One value of regression models is that these can reduce such bias. When a researcher uses regression approaches appropriately, these methods can provide valuable insights into the data. In their critique of a 2007 meta-analysis of circumcision and HPV by Van Howe, Castellsagué et al. pointed out that,* “Inexplicably, Van Howe fails to report also the pooled estimate of the crude OR. Instead he reports a meta-regression OR (adjusted by circumcision ascertainment and failure to sample the penile shaft) that we were unable to reproduce” *[24].

### 2.3. Flaws in the Search Strategy

Van Howe's article failed to include a number of publications, many of which were identified in previous meta-analyses. The search strategy used should have included, at the very least, combing previous meta-analyses for references to ensure completeness. The Cochrane Handbook, for example, points out in Section 6.2 that authors should check the reference lists of articles identified and of previously published reviews to help find relevant reports [25]. Following up references from previous articles often proves an efficient means to identify studies for possible inclusion in a review. As stated in its Section 10,* “because investigators may selectively cite studies with positive *results (our references [26, 27]), reference lists should never be used as a sole rather as an adjunct to other approaches" [25].

### 2.4. Omission of Studies That Met the Inclusion Criteria

The article selectively ignores some of the studies that it retrieved. Several relevant studies listed in Van Howe's Table 1 as ones that met the inclusion criteria do not appear in his tables of studies included in each meta-analysis of a particular STI (the article's Tables 3–14). Our table (Table 1) lists some of these missing studies. It is scientifically unjustified to ignore, without adequate explanation, studies that meet inclusion criteria.

### 2.5. Basic Statistical Naivety

A somewhat trivial criticism in comparison to our other concerns is failure to understand that the number of decimal places permitted in presentations of data is determined by the value of “*n*” (i.e., the size of the denominator) and the accuracy of the data measurement. Throughout the article ORs, CIs, *P* values, and other data are presented to 4, 5, or even 6 significant figures. In doing this, the figures presented exceed considerably, often by orders of magnitude, the number of significant figures in the source data used to generate these values. This practice moreover serves no useful purpose.

## 3. Flaws in Particular Methods Used

### 3.1. Apparent Manipulations of Unadjusted Results That Did Generally Support the Scientific Consensus

In general, Van Howe's initial meta-analyses found male circumcision to be protective against GUD, HPV, and HSV-2. The findings from these data assessments are consistent with findings from the meta-analyses performed by others. However, he then performed various adjustments, including removal of studies referred to as outliers (his Subsection 3.3) so as to* “bring the overall between-study heterogeneity to within an acceptable range *(*P* > .10)*.”* This results in the article ultimately making the claim that uncircumcised men are at significantly lower overall risk for an STI.

As an example of an overall impression of careless writing throughout, the article states, “*P* > .10” in Subsection 3.3, but “*P* < .10” in Subsections 3.4 and 2.1—that is, note, “>” versus “<”. Furthermore, on page 19 of Subsection 3.6 a nonspecific statement appears saying,* “Methods to determine the presence of publication bias use a P value threshold of 0.01 for significance”* (note* “0.01”* here not* “0.1”*).

### 3.2. Overstated Adjustment for “Lead-Time Bias”

Van Howe's Methods (Section 2 on page 2, column 2, paragraph 3 of the article) states, “*The three randomized clinical trials of adult male circumcision in Africa failed to adjust for lead-time bias*” and the Results, Subsection 3.2 entitled* “Meta-Analysis Results” *states in lines 2–4,* “when adjusted for lead-time bias, no statistically significant differences were noted in GDS, gonorrhea, syphilis, or any STI.” *Such a criticism of the trials has been well refuted previously, both in relation to the findings for HIV [67] and to those for HSV-2 [68], and would similarly lack validity when considering findings for other STIs.

The* “lead-time bias”* definition used in his article refers to men in the circumcised arm of these trials as having a shorter period of exposure to STIs over the 21–24 months of the trial because of advice to refrain from sexual activity during the healing period of 6 weeks following the operation. Six weeks was the period by which wound healing was certified as complete in 95.8% of men [68]. It is plausible and Van Howe is actually arguing that the difference seen between trial arms might be exaggerated, rather than being wholly attributable to such a bias. The problem is that, since his study lacks the original data set, his adjustments must of necessity be quite precarious and are based on assumptions that may or may not be true. The article does not explain the method used, but if, for example, it involves simply multiplying the number of cases in the uncircumcised group by a correction factor, then it is implicitly assuming that the rate of new infections was constant over time. That may not be the case. The effect of 6 weeks abstinence from sexual intercourse would be modest (1.5/21 months = 6.6% for the trial of 21 months [69] and 1.5/24 months = 5.8% for the trials of 24 months [70, 71]) and could not account for the magnitude of the difference in STI incidence between men in the intervention and control arms of each trial. The Johns Hopkins group has reported a strong protective effect against HPV in both the 0–12 and the 12–24 month periods after circumcision [32].

In the case of findings for HSV-2 in the Ugandan trial, Tobian et al. show in their Figure 2 that the difference between circumcised and uncircumcised men becomes greater with time [28] (Van Howe's reference [82]). If these results were affected by* “lead-time bias,”* then the difference in incidence between the circumcised and uncircumcised groups should have been the same at 12 months and at 24 months after commencement of the trial. Tobian et al. provided the necessary calculations, showing that,* “adjusting for this period of sexual abstinence, we estimate that the incidence of HSV-2 infection would be 4.2 per 100 person-years (114 of 2714 person-years) in the intervention group and 5.4 per 100 person-years (153 of 2851 person years) in the control group *(*P* = .02)*”* [68]. Thus, the data show that circumcision has a significant protective effect when* “lead-time bias”* is taken into account. This particular quote by Tobian et al. [68] was in fact a response to criticisms by Van Howe's spouse, Michelle Storms [72] (Van Howe's reference [26]), where, interestingly, Storms makes the “lead-time bias” accusation against Tobian et al.'s HSV-2 data but does not raise this issue in relation to that paper's HPV data. Van Howe's 2013 article ignores the response by Tobian et al.

### 3.3. Inappropriate Adjustment for “Sampling Bias”

The studies of HPV in circumcised and uncircumcised men involved sampling from a single site or multiple sites on the penis (Table 2).

The method his article uses to adjust for sampling bias is one that was devised in 2007 [73], elaborated on in a later paper [74] and referred to elsewhere [75]. His article states, “*The effect of sampling bias has been consistent in the medical literature,*” yet it then cites only Van Howe's own 2007 meta-analysis [73]. His method has been criticized by several different groups of researchers [24, 68, 76], but his article ignores such criticisms.

In the earliest critique, in 2007, Castellsagué et al. pointed out, “*The second serious mistake is his approach of manipulating the original published data by applying HPV detection rates from one study to other studies to compensate for supposed HPV under-detection caused by not including samples from the penile shaft*,” going on to say, “*Whether or not the penile shaft and scrotum should or should not be sampled is still an open and debated scientific question*” [24].

Van Howe's “*sampling bias*” issue comes from a critique by Travis in 2002 of Castellsagué et al. [77], in which Travis stated that the glans, “*surface is dry on circumcised penises but moist on intact penises, increasing the likelihood of detection of HPV regardless of the actual rate of infection*” [78]. In response to Travis, Castellsagué et al. wrote, “*Although it is plausible that circumcision compromises cellular yield, the quality and sensitivity of our polymerase chain reaction overcome this potential limitation. We used amplification of a fragment of the *β*-globin gene as an internal quality control for each specimen, thus ensuring both the high quality of the DNA and the presence of cells. Samples from which the *β*-globin and HPV L1 genes could not be amplified were excluded from the analyses, and no differences were found between the subjects with such samples and those with valid samples*” [79]. The response by Castellsagué et al. succinctly answers the objection on page 31, column 1, paragraph 4 of Van Howe's article where he states, “*There is also the question of whether the glans of the circumcision *[*sic*]* is too dry to allow for accurate sampling.*” His article ignores this quite reasonable explanation.

The* “sampling bias”* allegation has also been made against the large RCTs evaluating the effect of male circumcision on STIs [74]. The authors of one of the trials to report data for HPV, Tobian et al., in a response to a similar criticism by Storms [72], stated that they were aware that,* “circumcised men had less HPV on the glans penis than uncircumcised men,”* but then went on to say,* “we do not agree that this is sampling bias,”* arguing instead that, “*it is a biologic effect of removing the foreskin*” and not a sampling effect, stating that Storms',* “adjustment for the biologic effect of circumcision on HPV conflates cause and effect and results in an uninterpretable underestimate of efficacy”* [68]. In a later paper that addressed the anatomical sites issue specifically, Tobian et al. found, “*the point prevalence of any HR-HPV infection at the one year visit on the coronal sulcus was lower in the intervention men (21.5%) than control men (36.3%)*” and “*The point prevalence of any HR-HPV infection on the shaft at year one was lower in the intervention arm (15.5%) than control arm (23.8%)*” [29]; that is, the protective effect on the glans/sulcus (41%) was similar to that on the penile shaft (35%). The finding by Tobian et al. that *β*-globin is significantly lower among glans/coronal sulcus samples of circumcised compared with uncircumcised men [28] led these authors to conclude that their* “analyses may underestimate the protective effect of male circumcision*” [30].

In a 2009 critique [74] Van Howe accused Auvert et al. [31] of* “failing to consider”* sampling bias. But this is false. Auvert et al., who sampled from the urethra, not the glans/sulcus, had not only considered this potential source of bias but had conducted an experiment to test for it [31]. Nevertheless, in their reply, the authors of this South African trial proposed that the foreskin “*presents an inner mucosal surface vulnerable to HPV infection,”* and that,* “HPV acquired via the foreskin during sexual intercourse can be subsequently transmitted to the glans and corona sulcus on contact*” by* “autoinoculation” *[76]. Autoinoculation between anal canal/perianal region, perianal region and scrotum, scrotum and shaft, and shaft and glans/corona has been suggested as the reason viral load is similar in adjacent sites that come in close contact [60] (Van Howe's reference [106]). But HPV detection in more distal sites, such as the urethra, did not correlate. This might be regarded as one reason why the urethra might be seen as a desirable sampling site, although against this is the low prevalence of HPV in urethral samples as compared to other sites on the penis and the discomfort to the subjects when sampling from the urethra. Auvert et al. pointed out that they* “chose the urethral site for the detection of HR-HPV specifically because there was no anatomical reason that could explain a differential effect of circumcision status on the detection of HPV or associated lesions at this site”* and “*The urethra was chosen because the detection of HPV in this anatomical site is probably not affected by circumcision status*.” Van Howe's article does not acknowledge any of this.

Auvert et al. described the nested study they conducted that involved urethral swab sampling before and after circumcision to exclude the possibility of sampling bias, stating,* “To ascertain that the detection of HR-HPV was not affected by circumcision status, we used these swab samples to compare the prevalence of HR-HPV among the nested study participants before and after circumcision*” (see page 15, column 1, of their article). On page 17, column 1 of their paper Auvert et al. present the findings for 371 men who underwent urethral swab sampling before and after circumcision, finding high-risk HPV to be* “23.7% versus 23.9%”* for sampling before circumcision versus sampling a median of 43 days after circumcision and infection by multiple high-risk HPV to be* “10.2% versus 12.1%.”* Auvert et al. concluded,* “These results indicate that the as-treated effect of MC on HR-HPV prevalence shown *[in Table 2 of their paper]* cannot be attributed to easier detection of HR-HPV by urethral swab sampling in uncircumcised men*.” In short, Auvert et al. convincingly excluded* “sampling bias.”* Despite this, Van Howe's article ignores their response [76], citing instead the* “sampling bias” *assertion [74] as though it remains valid.

As also pointed out by Auvert et al., “*the prevalence of HR-HPV infections in our cohort is likely underestimated, because the rate of detection in the urethra is significantly lower than that in the glans, corona sulcus, or penis shaft.*” They then go on to say, “*we believe that HR-HPV infections would be underestimated equally in the 2 arms and that this underestimation would have no effect on PRRs *[prevalence risk ratios]” [31]. Thus the issue of “sampling bias” was addressed by these trial authors and was not supported by the evidence they provided. We suggest that Van Howe has either not examined this paper or has chosen to ignore key data that conflict with the argument he presents.

An earlier study in the USA and Mexico, cited by Giuliano et al., found HPV to be the highest on the shaft (48%) and glans penis/coronal sulcus (33%), but to be lower for urethral sampling (10%) [35]. Aguilar et al. also cited a study of Mexican soldiers that found HPV prevalence to be higher on the skin of the external genitalia than in the urethra [34]. These publications are not cited in Van Howe's article.

Auvert et al. concluded by saying, “*The protective effect *[of circumcision seen in their trial]* corresponds in magnitude to what could have been expected from observational studies*.”

In the Discussion (page 31, column 1, paragraph 3) Van Howe's article states, “*There are only two reasons for the Johns Hopkins researchers to withhold the evidence they collected; either they were not current on the medical literature as it applied *[to]* the research they were conducting and reporting or they purposely withheld results of the swabs taken from the penile shaft*.” His article then concludes by saying, “*Basically, when Tobian et al. and Auvert et al*. [where Auvert is in Paris, France, not Baltimore, Maryland, USA]* reported only on sampling from the glans, they guaranteed a positive finding because the location of HPV on the penis differs according to circumcision status,*” citing the following references: [28, 31]. Van Howe seems unaware that, in addition to their 2009 publication in the* New England Journal of Medicine*, the Johns Hopkins group subsequently published all of their data in several other articles, namely, Gray et al. in 2010 [32] and Tobian et al. in 2011 [29] and 2012 [30]. These evaluated HPV in the men at enrolment and then at 6, 12, and 24 months. The paper by Gray et al. stated, “*We collected swabs from the coronal sulcus/glans and the shaft, but only had resources to assay the corona sulcus/glans samples*” [32]. Data for HPV on the shaft (and coronal sulcus) were published in 2011 by Tobian et al. [29]. The paper by Tobian et al. in 2012 sampled only from the coronal sulcus. These later papers by the Johns Hopkins group invalidate Van Howe's speculative assertion and outrageous, incorrect, unprofessional, and entirely inappropriate statement that, “*Neither of the options, incompetence or willful [sic] academic misconduct, is appealing*” (page 31, column 1, paragraph 3). This is also an example of the false dichotomy fallacy. A third option is that the researchers being accused have a better understanding of how to sample for HPV than is apparent in Van Howe's article. His article fails to acknowledge the published explanation (above) by the researchers that, at the time, they simply did not have the resources to test all the samples. Thus it would do well for Van Howe to read the articles he criticizes.

In his 2007 meta-analysis of circumcision and HPV [73], Van Howe based his adjustment for* “sampling bias”* on data in a paper by Weaver et al. [59] that obtained penile shaft, glans, foreskin, and scrotum samples, as well as urine from 318 male university students. But the number of infected circumcised and uncircumcised men in the study by Weaver et al. was too small to provide reliable data supporting the kinds of calculations Van Howe's 2007 meta-analysis adopted, since they were in the range of experimental variation. The Weaver study found HPV in 82 circumcised men and 17 uncircumcised men. On page 681 of that paper, Weaver et al. state,* “Of the 82 circumcised men who were found to be positive for HPV DNA at any genital site, 63 (77%) had HPV DNA detected in the sample from the penile shaft, 39 (48%) had HPV DNA detected in the sample from the glans, 43 (52%) had HPV DNA detected in the sample from the scrotum, and 13 (16%) had HPV DNA detected in the urine sample.”* This is where Van Howe's 2007 meta-analysis obtained the figure of* “48%”* for the glans for that article's* “sampling bias”* adjustment for circumcised men. The figure of “65%” for uncircumcised men came from the statement by Weaver et al.,* “uncircumcised men who were found to be positive for HPV DNA at any genital site, 9 (53%) had HPV DNA detected in the sample from the penile shaft, 11 (65%) had HPV DNA detected in the sample from the glans, 15 (88%) had HPV DNA detected in the sample from the foreskin, 9 (53%) had HPV DNA detected in the sample from the scrotum, and 5 (29%) had HPV DNA detected in the urine sample.” *The small *n* values on which Van Howe's 2007 meta-analysis based its adjustments could be regarded as reflective of statistical naivety at best and thus a further demonstration of the questionable nature of the data generated by his 2007 meta-analysis. Of the 17 uncircumcised men who were HPV-positive, only 11 had HPV on the glans. While this represents 65%, if the proportion had been the same as was seen in circumcised men of 48%, the number would have been 8. The difference, (11 minus 8) is just 3 individuals. Similar calculations for the shaft yield just 4 individuals. The adjustments could thus have been affected by just 3 or 4 individuals! These sample sizes are too small to base sampling bias adjustments on.

The figures reported by Weaver et al. for the scrotum that Van Howe's 2007 meta-analysis chose not to show were similar for circumcised and uncircumcised men, whereas those for the shaft and urine were very different.

Thus random variations based on inadequate sample sizes had the potential to contribute to critical calculations, rendering the adjustment unreliable. Meta-analyses are intended to improve precision, not only by effectively increasing sample size, but also by diluting the effects of individual study biases. By using the results of a small study to manipulate data in the other studies, Van Howe's article has jettisoned precision by effectively giving the imprecise data from those small studies a great deal of weight in the adjustment. Although a somewhat more sensible approach might have been to calculate a CI for the correction factor and then perform the analysis separately using both bounds of this interval, the whole concept of applying such a correction is bewildering.

In fact, Weaver et al. concluded that, “*Interestingly, the HPV DNA prevalence for circumcised men was similar to that for uncircumcised men (31% [82/258] versus 29% [17/59]; P* = .66)” [59]. Van Howe further fails to mention that Weaver et al. question whether *β*-globin DNA needs to be present given that some samples were negative for *β*-globin DNA, but positive for HPV. Weaver et al. noted that, “*Testing multiple sites increased the likelihood of detecting HPV DNA, especially among circumcised men. We observed similar percentages of HPV DNA positivity among circumcised and uncircumcised men.*” In discussing other studies, Weaver et al. pointed out that sampling from the penile shaft “*appears to be critical for HPV testing, especially among circumcised men. In the present study, if only samples from the glans and foreskin had been tested, only 17% of circumcised men (39/233) and 32% of uncircumcised men (16/50) would have had HPV DNA detected, resulting in an overall prevalence of only 19% (55/283) and a significant difference in HPV positivity between circumcised and uncircumcised men. Thus, failure to obtain samples from other sites, such as the penile shaft and the scrotum, appears to substantially reduce the number of genital HPV infections detected among circumcised men” *[59].

That circumcision protects against HPV in the glans area seems reasonable given that the glans area is the site of the most obvious difference—being exposed in circumcised males, but covered in uncircumcised ones. Perhaps coverage by the foreskin provides a more favourable habitat for the virus or facilitates transfer from glans to inner foreskin (or* vice versa*) and back again so leading to reinfection or spread of infection. Sampling only the shaft and scrotum would miss the protective effect in the glans area, just as sampling only the glans area would lead to an inflated measure of overall penile protection. Sampling multiple areas would give a better overall measure of protection.

In the last paragraph of their paper, Weaver et al. say, “*for HPV screening of circumcised men, we recommend that multiple sites–including the penile shaft, glans, coronal sulcus, scrotum, and urine be tested,” *and go on to state*, “For HPV screening of uncircumcised men, we recommend that samples from the internal foreskin, which could be included with the glans and coronal sulcus in 1 sample, and from the penile shaft, including the external foreskin, be obtained for HPV DNA testing in this population. In the present study, testing additional sites, such as the scrotum and urine, did not yield an increase in HPV positivity among uncircumcised men” *[59].

In their critique, Auvert et al. use HPV data for the shaft (Van Howe's favoured sampling site), finding, “*According to Van Howe's method, the adjustment of our results for this difference in yield reveals a statistically significant protective effect of a magnitude greater than that reported in our study,*” so leading Auvert et al. to state, “*Hence, it is clear that, if there is a sampling bias, it is not unidirectional, unlike what Van Howe argues*” [76].

In the Methods section (page 2, column 2, paragraph 6, lines 5–8) Van Howe's article states, “*To adjust for the impact of this sampling bias, separate analyses were performed by multiplying the number of infections identified in studies that only sampled the glans by 1.514 in intact males and 2.212 in circumcised males.*” Instead of using data from the study by Weaver et al. [59] to calculate the adjustment factors, Van Howe uses data from a larger follow-up study by the same group in Seattle of 477 male university students, from whom “*shaft/scrotum, glans, and urine samples were tested for 37 alpha HPV genotypes*” [39]. He cites this study by VanBuskirk et al. in lines 2–4 of Methods paragraph 6 by stating, “*if only the glans is sampled, only 66.1% of the intact men with genital HPV would be identified, while only 45.2% of the circumcised men with HPV would be identified*” [39] (Van Howe's reference [32]). Our inspection of that paper shows that the figures of “66.1%” and “45.2%” for the “corrections” applied in Van Howe's meta-analyses article are derived from data in Table 4 of VanBuskirk et al. [39]. These show that for circumcised men HPV was detected on the glans of 100 + 1 + 120 + 5 (glans only, urine and glans, glans and shaft, and all sites, resp.) = 226 of 500 infections (45.2%), and for uncircumcised men HPV was detected on the glans of 51 + 0 + 45 + 13 = 109 of 165 infections (66.1%). Since these are for sampling of the glans only (as Van Howe's article acknowledges in the first quote above), they cannot be used as “corrections” to data from studies that used data from other areas, such as the urethra, sulcus, and shaft. In fact, if the data of VanBuskirk et al. for “shaft/scrotum” had been applied, the “bias” would go in the opposite direction: circumcised 378/500 (75.6%) and uncircumcised 107/165 (64.8%) [39]. It would thus be invalid for Van Howe's article to apply its “correction” to data that did not arise from sampling the glans.

Van Howe's Subsection 3.5.5 states, “Studies that sample only the glans had a summary odds ratio of 1.86 (95% CI = 0.9964–3.46), while studies with complete sampling had a summary odds ratio of 1.10 (95% CI = 0.88–1.37).” There is a well-known ascending gradient of high-risk HPV types towards the tip of the penis. It is the glans underlying the foreskin of an uncircumcised man that is a common site of penile lesions leading to cancer. In view of the more aggressive collection method used by VanBuskirk et al. (rubbing the genital skin sites with emery paper to loosen up cells so as to yield 45% *β*-globin DNA compared with 23% using a wetted Dacron swab [59]), the sampling method is unlikely to be problematic. VanBuskirk et al. found an actual difference in HPV distribution across the penis between circumcised and uncircumcised men, not a “sampling bias.” These authors did not, however, test for HPV in the urethra. It therefore seems even more inappropriate for Van Howe to apply his “correction” to data that had arisen from urethral sampling, such as was used in the RCT by Auvert et al. [31].

Another issue is whether one can extrapolate data from use of one sampling method (such as emery paper) to data from use of other methods of sampling (such as urethral swabs or visual inspection). This is all the more applicable considering the differences observed when using different methods. Weaver et al. stated that,* “emery paper (600-grit Wetordry Tri-M-ite) and a saline-wetted Dacron swab were used for collection of cells.” *As mentioned above, they found 45% of emery paper and 23% of Dacron swab samples to be positive for *β*-globin DNA, which is a marker for adequate collection of cellular material used for HPV testing [59]. VanBuskirk et al. stated,* “Exfoliated epithelial cells from genital sites were collected by first using emery paper (3 M) to loosen cells and then pre-wetted Dacron swabs (E.I. du Pont de Nemours) to collect them.”* In this study it was the uncircumcised men that tended to give inadequate samples, with VanBuskirk et al. stating,* “While sample insufficiency was low at all sites, uncircumcised men were more likely than circumcised men to have insufficient glans (1.92% versus 0.74%, P* < .01, *Z-test) and shaft/scrotum samples (1.28% versus 0.46%, P* < .01, *Z-test).”*

The studies by Gray et al. in their 2010 paper and Tobian et al. in 2011, which did not use emery paper, found that circumcised men tended to give insufficient samples at the sulcus. We therefore invoke an additional concern, namely, whether it is valid to correct glans data using a “correction” derived from use of the emery paper method of VanBuskirk et al. to data obtained by Dacron swabs used by Gray et al. and Tobian et al. What seems nevertheless to be abundantly clear is that Van Howe's “correction” is certain to be inapplicable to data arising from urethral swabs or, as will be discussed below, visual examination of penile lesions. Neither Weaver et al. nor VanBuskirk et al. sampled from the urethra. Yet Van Howe applies the “correction” to the RCT data of Auvert et al. who only sampled the urethra. VanBuskirk et al. did sample urine, however, and state that, “*urine sample insufficiency was comparable for circumcised and uncircumcised men (0.49% versus 0.37%, P* = .13, *Z-test)*” and “*Insufficiency was 0% when results from all three sites were pooled.*” In addition they stated, “*Incident type specific infections first detected in the glans and/or urine only were 2.7 (95% *CI*: 1.6–4.5) times more likely to occur in uncircumcised men than those detected in the shaft/scrotum only. Incident infections detected in both the shaft-scrotum and the glans and/or urine were 2.4 (95% *CI*: 1.5–3.9) times more likely to occur in uncircumcised men than infections detected in the shaft/scrotum only.*” These authors provided no indication of being aware of the “*sampling bias*” adjustment. They were, however, familiar with the concept of performing adjustments to data, doing so for number of new sex partners. Despite the attention Van Howe's article gives to the paper by VanBuskirk et al., it did not include the latter in the meta-analysis he performed.

It should thus be apparent that studies looking at HPV on one area only—be it glans, urethra, or elsewhere—are only looking at the effect of circumcision on HPV in or on that area. Since the Van Howe's correction fails on two counts—different sampling site and different sampling method—the data arising can be dismissed completely.

Two different RCTs have now demonstrated that male circumcision reduces HPV at the urethra, coronal sulcus, and the penile shaft [29, 31, 68], clearly removing the concern about* “sampling bias”* and providing further biological plausibility. It has also been demonstrated recently that male circumcision reduces HPV load [80].

### 3.4. Inappropriate Adjustment for “Publication Bias”

To assess publication bias Van Howe generated a*“funnel graph of precision (1/variance) by the natural logarithm of the odds ratio”* for each STI (Figures 6–16 in his article), using linear regression analyses, funnel plot regression, and the adjusted rank correlation test to demonstrate the degree of between-study heterogeneity, so justifying the article's decision to remove* “outlier studies.”* The results for each STI by each of the 6 methods used to adjust for publication bias show that based on* “a P value threshold of .10 for significance”* (Subsection 3.6, page 19, last paragraph) 22 of the 72 analyses presented in the article's Table 17 are significant. It concludes that,* “Of the six measures of publication bias, none were positive for GUD, syphilis, and genital warts; one was positive for chlamydia, gonorrhea, HSV, and any STI with the study of Langeni* [our reference [81]]* excluded; three were positive for NSU and HPV; and four were positive for GDS and any STI with Langeni* [our reference [81]]* included.”*

The article then adopts a* “trim-and-fill”* approach to adjust for publication bias, justifying the removal of certain studies. A weakness of the* “trim and fill”* method is that it assumes there is a publication bias. It fails to account for the possibility that there is no bias or that asymmetry in the funnel plot could be due to something else. As a result Van Howe's article may be introducing an* unnecessary* “correction” into the analyses. To quote from the* “Trim and Fill”* Section (10.4.4.2) of part 2 of the* Cochrane Handbook for Systematic Reviews of Interventions* [25], “(the trim and fill method)* is built on the strong assumption that there should be a symmetric funnel plot, and there is no guarantee that the adjusted intervention effect matches what would have been observed in the absence of publication bias, since we cannot know the true mechanism for publication bias. Equally importantly, the trim and fill method does not take into account reasons for funnel plot asymmetry other than publication bias. Therefore, “corrected” intervention effect estimates from this method should be interpreted with* *great caution. The method is known to perform poorly in the presence of substantial between-study heterogeneity. Additionally, estimation and inferences are based on a dataset containing imputed intervention effect estimates. Such estimates, it can be argued, inappropriately contribute information that reduces the uncertainty in the summary intervention effect.” *An examination of Table 15 of Van Howe's article reveals that there is significant heterogeneity in just about every analysis, suggesting that the “trim and fill” approach is unsuitable.

Interestingly, Van Howe stated on page 485 (top right) of his 2007 meta-analysis of HPV that, “*Adjustment for publication bias needs to viewed with caution as asymmetry of a funnel plot may be due to factors other than publication bias, and, likewise, results generated to correct for the asymmetry may not reflect a correction for publication bias*” [73]. Manipulation of data can change the appearance of a funnel graph. One might, for example, cause a point to move to the left (the direction of harm from circumcision), or one might take a point already to the left and move it up (to give it more weight).

Van Howe's article makes so many adjustments that some readers may become confused and end up relying simply on its show of “expertise.” The question that can be reasonably posed is whether the manipulations actually represent a scholarly effort intent on arriving at impartial findings or whether they are part of a contrived attempt to reach a predetermined conclusion.

### 3.5. Inappropriate Manipulation of Control Groups. 

Van Howe's “Methods” Section (page 2, column 2, paragraph 2) states, “*several older studies had inappropriate control groups*” [82–84], going on to say, “*For example, Hand used men without any exposure to STIs as controls,*” and later that, “*Wilson compared seasoned soldiers with new recruits*” (page 33, column 2, paragraph 2). Taking the latter as an example, the article rejects Wilson's control group and instead compares men with a particular STI with all men in the STI population. In so doing the article implicitly assumes that circumcision has no effect for all other STIs.

In a critical analysis of Van Howe's meta-analysis in 2007 of sexually transmitted urethritis [62], Waskett et al. noticed that the ORs reported by Van Howe did not accord with the literature [22]. In their critique they presented the results of a meta-analysis of one of these, NGU, for the same 10 studies employed in Van Howe's 2007 meta-analysis, but used instead the original source data for the circumcised and uncircumcised case and control groups (see their Table 2). They found that, rather than the summary OR being 0.81 (95% CI 0.64–1.01) as reported by Van Howe, the OR was 1.10 (95% CI 0.78–1.55) [22]. (Note that ORs were expressed in an inverse manner in that 2007 article compared to the present 2013 article.) Our table (Table 3) expands on Table 1 in Waskett et al. by including data for the four studies highlighted by Waskett et al. in their critique. Thus use of the actual data showed a stronger trend towards circumcision affording a protective effect against NGU, although this was not statistically significant.

In a response to the* critique* by Waskett et al., Van Howe admitted that, “*the data for the study by Cook et al. was *[sic!]* improperly extracted (the “Reference Group” was interpreted as being the number in the study)*” [85]. His reply referred to the studies by Cook [63] and by Parker [66], that used as controls men who did not have a STI, and stated that the study used “*all of those who sought care at these sexually transmitted disease clinics who met the inclusion criteria*” and “*disease rates were compared in men with and without a certain trait.*” Van Howe's 2013 meta-analyses article states explicitly that the control groups were modified—see his Methods section (page 2, column 2, paragraph 2, lines 5–8): “*In an attempt to control for exposure to STIs, men with a particular STI were compared to all men presenting for evaluation for the possibility of an STI*.” Since the tables provide the frequencies being used for each group, we have been able to deduce how figures at odds with original studies were obtained. We will use the study by Cook [63] as an example. In Van Howe's Table 7, the figures shown for the cases (87 and 175 for uncircumcised and circumcised men, resp.) match those of Cook. However, the figures shown for the control group do not (198 and 787 for the respective groups in Cook's paper versus 453 and 2061 in Van Howe's Table 7). Since we now know that the method Van Howe adopted was to use as a control group all men who did not have gonorrhea, by calculating the total number of men in the study (87 + 453 = 540 uncircumcised men, and 453 + 2061 = 2236 circumcised men) and then subtracting the number of men who had gonorrhea (i.e., 540 − 87 = 453 for uncircumcised men and 2236 − 175 = 2061 for circumcised men), we obtained the figures for the control group of uncircumcised men (453) and circumcised men (2061) that appear in Van Howe's Table 7. Irrespective of whether one might agree with the method Van Howe uses, at least it is now clear how some of his figures were derived, so further explaining why various ORs shown in his Tables differ from the ORs reported in the original study.

In light of the fact that the calculations in Van Howe's article create data for a different control group than that used in the original studies, Van Howe should have also conducted a meta-analysis of the original source data that appeared in the studies he used. By essentially redesigning studies after the fact, his article fails to perform a meta-analysis of the existing literature, thus risking injection of biases.

## 4. Meta-Analysis Results for Individual STIs

We will now address the meta-analyses of each specific STI, starting with HPV. Since Van Howe's article devotes the most attention to HPV, its claims about circumcision and HPV deserve the most wide-ranging and extensive scrutiny.

### 4.1. Human Papillomavirus

In Subsections 3.5.5 of Van Howe's Results and 4.11 of his Discussion, circumcised men were found to have a reduced risk of HPV across all studies (random effects summary effect OR for* “Any HPV”* = “1.2411”; 95% CI “1.02–1.51”). However, the article then goes on to conduct separate analyses of studies of* “high-risk HPV”* and studies using his preferred sampling method* ("selective HPV"*), resulting in random effects summary effect ORs of “1.1661” (95% CI “0.94–1.45”) and “1.0128” (95% CI “0.80–1.1”), respectively. The* “high risk HPV”* meta-analysis included only 4 of the 20 studies listed in his Table 13 (excluding data from studies that grouped high and low risk HPV genotypes together). The footnotes to Table 13 suggest that his analysis overlooked inclusion of the “#” symbol against* “Vardas, high risk”* [86] as being one of the studies of high-risk HPV used to calculate a “*high risk HPV summary effect.”* Thus his analysis should have included 5 studies. Even more odd is that the* “any HPV”* analysis appeared to include only 4 studies again, apparently owing to the same problem with mistakenly excluding Vardas et al. [86]. It seems that an attempt was being made by Van Howe to make this particular table look impressive by listing data from a considerable number of various relevant studies shown in his Table 1 entitled* “Attributes of all studies meeting the inclusion criteria.”* However, by failing to include the majority of studies, most of the data have been discarded.

Curiously, two RCTs of high-risk HPV listed in Van Howe's Table 1 [28, 31] do not appear in his Table 13. The reason is not specified. This omission is surprising since RCTs are regarded as providing high quality evidence. Even more extraordinary is that another high quality RCT [32] appears neither in the article's Table 1 or Table 13. Instead this paper (reference [83] in his article) is only cited fleetingly in two places: on page 3, right-hand column, paragraph 2, lines 22–24, next to* “Some studies have looked at clearance rates of HPV from the penis, but these were not part of this analysis,”* and near the end of Subsection 3.1 in relation to* “clearance rates of HPV.”* Van Howe thereby demonstrates an awareness of the existence of this publication, whose title refers to HPV acquisition. These oversights are surprising since those RCTs formed part of a 2012 meta-analysis of circumcision and HPV by Albero et al. [87] that Van Howe's article cites in its Introduction and again in its Discussion along with a review (not a meta-analysis) by Rehmeyer in 2011 [88]. Both of these contradict the findings from Van Howe's 2007 meta-analysis [73]. The meta-analysis by Albero et al. included data from other studies as well [36–38], yet these are omitted by Van Howe without explanation. Failure to include data from the RCTs cited, data from a RCT that is not cited [89], and data from other studies should raise further doubts about the validity of the results of his meta-analysis. One would have expected RCT data in particular to be of high quality, thus meriting its inclusion in a meta-analysis aimed at* “calculating *[a]* high risk HPV summary effect.”*

In the trial in Uganda by researchers from Johns Hopkins University men were randomized to circumcision or to the uncircumcised control group and tested for HPV at baseline and 24 months after circumcision of the intervention group, and for HIV, HSV-2, and syphilis at baseline and 6, 12, and 24 months [28]. HPV was detected in samples collected from the preputial cavity of uncircumcised men and the coronal sulcus of circumcised men (this information appears in the online supplement accompanying reference [28]). Tobian et al. were careful in pointing out that,* “these subjects may represent a self-selected population of compliant subjects who could be at lower risk for HPV infection than the general population; this factor could result in an underestimation of the efficacy of male circumcision.”* They were also cautious in saying, “*since samples were evaluated only at 24 months, we were limited in our ability to determine whether the reduced HPV prevalence after circumcision was due to a reduced rate of HPV acquisition, an increased rate of HPV clearance, or both*.”

Van Howe's article considers why, if clearance of a HPV infection takes longer for uncircumcised men, is HPV prevalence is not higher in such men. The other issue is viral load. Prevalence and viral load are two different types of data, one dichotomous and the other continuous. One might expect that rate of clearance would be associated with load, not necessarily prevalence. On page 31 (column 2, paragraph 2) Van Howe bemoans a letter to the editor that,* “the editor refused to publish.” *His rejected letter apparently criticized an RCT in Kenya [33] (Van Howe's reference [77], cited in his Section 3.1 as being, without adequate explanation, amongst* “Several studies* [that]* had collected the data that would have met the inclusion criteria but did not report their results in a manner to include them in the analyses”*). The letter apparently asked,* “that the authors provide the results of the incidence of *[while saying earlier* “overall rates of”*]* HPV infection by circumcision status.”* Van Howe states,* “If one back calculates using the rates of infections by the type of penile lesion and rates of the types of lesions by circumcision status and assumes there is no interaction between these factors, there is no statistically significant difference between HPV infection rates based on circumcision status.”* The statement probably meant to say “prevalence,” not “rates.” The back calculations are at best overly naïve and simplistic and fail to take into account other factors that affect HPV prevalence, the pathological significance of the anatomical sites where infection occurs, and from where transmission takes place, amongst others.

Van Howe's discussion of HPV clearance is inadequate. On page 31 (column 1, last paragraph) it states,* “A couple for* [sic]* studies have indicated that the clearance of HPV takes longer from the intact penis [35, 55, 83, 84],”* the latter being of course four (not* “A couple”* of) references cited in support. (Van Howe's references [35], [55], [78] and [79] correspond to references [69], [30], [32] and [37], respectively, in the present article.) His reference [35], a study of HPV clearance in men at the University of Hawaii, reported that,* “a lower percentage of sufficient glans/coronal sulcus samples among circumcised men would make detection of a clearance event less likely, which may therefore lead to a longer estimated duration of HPV infection among circumcised men. However, the opposite was observed in our data—that is, circumcised men had a shorter duration of infection of the glans/coronal sulcus”* [61]. In contrast, “*The duration of infection did not vary by circumcision status in the penile shaft, scrotum, or all genital sites combined.”* Thus clearance is greatest in precisely the area of the penis exposed by circumcision. This study concluded, moreover, that,* “the higher prevalence of HPV may be attributed to a longer duration of infection of the glans/coronal sulcus among uncircumcised men rather than to a greater rate of acquisition of infection.”*

A study in the USA (Van Howe's reference [84]) sampled the glans/corona, shaft, and scrotum by swab and reported combined results showing, “*Clinically confirmed circumcision was significantly associated with an increased likelihood of clearance of any HPV infection (HR, 2.7 *[95% CI, 1.3–5.7]*) and of clearance of oncogenic HPV infection (HR, *3.2 [95% CI, 1.4–7.4]*), but not with an increased likelihood of clearance of nononcogenic HPV infection*” [37]. The authors concluded, “*Circumcision was most strongly associated with clearance of any HPV infection and with clearance of oncogenic HPV infection.*”

The study by Gray et al. (Van Howe's reference [83]) found that circumcision reduced acquisition of both high- and low-risk HPV types, and that the rate of clearance was significantly improved by circumcision [32]. Circumcision reduces the risk of microtears during sexual intercourse and eliminates the moist space beneath the foreskin and, as these authors suggested,* “progressive keratinization of surgical scar may reduce the number of basal cells vulnerable to HPV infections over time.” *It should be noted that the authors were referring to the* circumcision scar* on the penile shaft, not the glans. In a later paper from this group, keratinization of the scar again was used to explain lower detection of *β*-globin in glans/sulcus swabs of circumcised men, with the statement, “*this could bias the estimate of efficacy towards the null,*” that is, could lead to an underestimate of the efficacy of circumcision in protecting against HPV acquisition [29] (Van Howe's reference [104]).

Van Howe's article does consider the issue of faster HPV clearance in circumcised men, saying, “*If this is true, it is unclear what the clinical impact would be.*” Unfortunately, subsequent speculations miss the obvious clinical point that an uncircumcised man will be infective to his partner(s) for longer, thereby exposing them to greater risk of HPV acquisition. Even if circumcision were to make no difference to the acquisition of HPV (i.e., the number of times the subject acquires an infection, as claimed by Hernandez et al. [61] VanBuskirk et al. [39]), the uncircumcised man is still more likely to be simultaneously infected with multiple HPV genotypes (and on different parts of his penis) at any one time [39]. Van Howe's article ignores these impacts. VanBuskirk et al. noted, moreover, that uncircumcised men are more likely to have HPV at multiple sites.

His article ignores the fact that besides sampling the shaft and outer foreskin (shaft samples) and glans/corona/inner foreskin (glans samples) at 24 months in the Kenyan trial, Backes et al. also performed visual inspection of the shaft, glans, and both inner and outer foreskin using a colposcope after application of dilute acetic acid [33], a technique used commonly to reveal the likely presence of high-risk HPV. Flat penile lesions were seen in 12% of men, the most common site for these being the foreskin (9.9%), followed by the frenulum (3.3%) and glans (2.6%). They stated,* “Circumcised men had a lower prevalence of flat penile lesions *(0.7%)* versus uncircumcised *(26.0%)*; adjusted odds ratio *(OR) = 0.02; 95%* confidence interval *(CI) = 0.003–0.1.* Compared to men who were HPV negative, men who were HPV DNA positive *(OR = 6.5; 95% CI = 2.4–17.5)* or who had high HPV16/18/31 viral load *(OR = 5.2; 95% CI = 1.1–24.4)* had higher odds of flat penile lesions”* [33]. The finding by Backes et al. of a 98% lower incidence of flat penile lesions in circumcised men provides an independent measure of HPV infection in those men and is obviously a measure unaffected by Van Howe's “sampling bias,” which we have criticized. Yet this important publication was not included in his meta-analysis.

The prevalence of flat penile lesions among uncircumcised men in the Kenyan trial (26%) [33] was only slightly higher than prevalence in The Netherlands (17%) [42]. In each case such lesions correlated with detection of high-risk HPV genotypes. The prevalence of HPV in the flat penile lesions (77%) [33] was similar to a figure of 72% for HPV in flat penile lesions in men in The Netherlands whose female sexual partners had cervical intraepithelial neoplasia [42]. Backes et al. pointed out that, “*Flat penile lesions were strongly associated with high-risk HPV infection and high HPV16/18/31 viral loads, especially in the glans, supporting findings from previous studies that HPV might play a role in their etiology” *[33]. Given this strong association, the presence of flat penile lesions could almost be used as a proxy for high-risk HPV infection. Since they were determined visually by colposcopic examination of the entire penis, they should not be subject to the problems Van Howe's article complains about, such as sampling from a dry as compared to a moist glans for circumcised versus uncircumcised men, respectively. Therefore, in our view, his article would have done well to consider the data on flat penile lesions because they provide an independent measure of HPV that is less likely to be affected by Van Howe's questionable “sampling bias” claim.

If, as claimed, circumcision was unable reduce HPV in men, then how can one explain why, as stated in Van Howe's reference [50], “*Circumcision of adolescent and adult men in a rural Ugandan population significantly reduced the prevalence and incidence of both low-risk and high-risk HPV infections and increased clearance of high-risk HPV infections in their female partners*” [90]. The prevalence of high-risk HPV after 2 years was 28% lower among women with circumcised male partners than women with uncircumcised partners. Incidence was also lower by 23% in this RCT. Wawer et al. concluded,* “Our findings indicate that male circumcision should now be accepted as an efficacious intervention for reducing the prevalence and incidence of HPV infections in female partners.”* The authors suggested that the reason was the* “decreased HPV detection at the urethra, coronal sulcus, and shaft” *[90], going on to cite Tobian et al. [28] and Auvert et al. [31] and reporting that,* “male circumcision reduced the prevalence of high-risk HPV by 34% at the urethra and 35% at the coronal sulcus”* [90]. Wawer et al. suggested that,* “If male circumcision does not affect the risk of recurrence or reactivation, we could have underestimated the reduction of new infections*.” The large multinational study by Castellsagué et al. reported that cervical cancer was 58% lower in monogamous women with a circumcised high-risk male partner, defined as one who had had ≥6 sexual partners and first intercourse before the age of 17 years, as opposed to a male partner who was high-risk by these criteria but who was uncircumcised [77].

Thus Van Howe's failure to acknowledge the importance of viral load, as well as the fact that uncircumcised men are 10 times more likely to have the same HPV type detected at multiple genital sites [39], which, as suggested by these authors, has implications for HPV transmission, undermines his arguments. The accumulated load of high-risk HPV on and under the foreskin near the tip of the penis and its subsequent transmission during sexual intercourse into the cervical os of the vaginal canal of a female sexual partner might explain why lack of male circumcision is associated with a significantly higher risk of cervical high-risk HPV infection and cervical cancer [5, 77, 91]. In the large multinational study by Castellsagué et al. (Van Howe's reference [99]) sampling from the urethra and glans/coronal sulcus led them to report HPV infection in 5.5% of circumcised men compared with 19.6% of uncircumcised men [77].

### 4.2. Genital Warts

Van Howe lists 14 relevant articles on this topic in his Table 12 and obtains a random effects summary effect OR for his meta-analysis of “0.8225” (95% CI “0.65–1.04”). The topic is discussed in Section 4.10 on page 29 of his article. As a quick check for articles that might have been missed we examined Table 3 of the 2011 meta-analysis article by Larke et al. [92] (their Table 3). This competing meta-analysis was not cited by Van Howe. The reason should become obvious. Under the section on genital warts in Larke et al. we found five studies not present in Van Howe's Table 12. These included two by Aynaud et al. [40, 41] and one each by Bleeker et al. [42], Hart [43], and Oriel [44]. With the exception of Hart et al., each found substantially lower prevalence of genital warts in circumcised men. Van Howe was aware of the two studies by Aynaud et al., and those by Bleeker et al., Hart, and Oriel, since all are listed in his Table 1 (*“Attributes of studies meeting the inclusion criteria*”). So why are these studies missing from Table 12? Based on a meta-analysis of 17 studies in Larke et al., this paper concluded that there was* “No effect of circumcision on genital warts” *and that this* “may in part be due to detection bias if genital warts are more commonly reported and/or detected in circumcised men.” *A similar conclusion was reached in a meta-analysis of genital warts by Albero et al. [87]. We surmise that Van Howe's 2013 meta-analysis of genital warts should not be taken seriously because it is undermined by being clearly noncomprehensive as a result of omission of studies that might perhaps have not been favorable to a particular agenda.

### 4.3. Nongonococcal “Nonspecific” Urethritis (NGU)

Van Howe's article lists 12 relevant studies in his Table 5 and discusses these in his Section 4.3. Given the inadequacies of Van Howe's previous meta-analysis on the topic of sexually transmitted urethritis [62], which was severely criticized [22], we were particularly interested in seeing how his new analysis fared. After adjustments, his article finds that uncircumcised men have lower prevalence of NGU than circumcised men (random effects summary effect OR = “0.76”; 95% CI “0.63–0.92”). This can be compared with a finding of “OR = 0.8, 95% = 0.64–1.01” for his 2007 meta-analysis of NGU that included 10 of the same studies.

Our examination reveals that Van Howe's 2013 article has not addressed the concerns raised in the critique by Waskett et al. of his 2007 meta-analysis [22]. Earlier we pointed out that Van Howe essentially redesigned various studies and thus performed a meta-analysis of a contrived version of the literature. In the 2009 meta-analysis by Waskett et al., use of the actual source data resulted in an OR of 1.10 (95% CI 0.78–1.55), indicating a nonsignificant trend towards a protective effect of circumcision against NGU [22]. One might therefore expect that a meta-analysis of the actual data from the 12 studies cited in Van Howe's 2013 meta-analysis of NGU would again show that there is no truth to the conclusion to his Section 4.3 that,* “these analysis indicates *[*sic*]* a fairly robust, significant association between lower prevalence of NSU in intact males.”*

### 4.4. Chlamydia Trachomatis

Table 6 in Van Howe's article presents the 15 studies he uses to derive a random effects summary effect OR of “0.9099” (95% CI “0.72–1.15”) for Chlamydia. These findings are discussed in the article's Subsection 4.4. In the case of Laumann et al. [65], a critique of Van Howe's 2007 meta-analysis by Waskett et al. stated,* “Laumann *et al.* do not state an *OR* for chlamydia, nor do they provide sufficient data to calculate an *OR*, yet Van Howe provides one”* [22]. What Laumann et al. presented in their Table 2 was an adjusted OR for each,* “criterion variable.”* Since Laumann et al. do not provide n values for circumcision and positivity for* Chlamydia*, but they do provide rates per 1000, Van Howe might have decided to scale up this rate to the sample size, thus deriving frequencies from which a crude OR could be calculated. This could lead to an erroneous result because of the likelihood, for example, that not all of the subjects might have answered this question, meaning that the denominator would be incorrect and the derived frequencies would not be precise.

Another problem is that the study by Diseker et al. [23] listed in the Van Howe's Table 6 involved both a cohort study and a case-control study. His article only cites the case-control data, even though cohort studies are regarded as being of higher quality. The single set of data included by his Table 6 for Diseker et al. shows no significant difference in Chlamydia between circumcised and uncircumcised men (random effects summary effect OR = “0.8803”; 95% CI “0.6382–1.2057”). Van Howe's Discussion (Subsection 4.4 on pages 24-25) says that by removal of* “two outliers”* the,* “summary odds ratio is 0.93 *(95% CI = 0.87–1.00)” compared with the* “random effects summary effect *OR*” *of “0.9099” referred to above (see Van Howe's Table 6).

Van Howe's Table 6 omits several studies, including two high quality studies of New Zealand birth cohorts [47, 48] (his article's references [51, 138]) that were listed in his Table 1 showing that these met the inclusion criteria, and an RCT [45] that was also cited in the article's Table 1 (his reference [171]), showing an awareness of the existence of these studies. The prevalence of chlamydial infections in the Christchurch study was 52%, being nonsignificantly higher in uncircumcised men (OR 3.0, 95% CI 0.88–10.4), possibly influenced by the low n value [47], whereas prevalence was 39% in the larger study in Auckland, which found higher Chlamydia prevalence in the uncircumcised group in the case of men aged under 21 years and in those aged 26–32 years, but lower prevalence in men aged 21–26 years, with the findings for circumcised and uncircumcised men being not statistically different overall [48].

Thus, once again, we conclude that his meta-analysis is inadequate.

### 4.5. Neisseria Gonorrhea

Van Howe's article draws on data from 23 studies in deriving a random effects summary effect OR of “1.0272” (95% CI “0.86–1.23”) for gonorrhea in his Table 7. The values for Cook et al. [63] in Table 7 are similar to those in his Erratum published in 2009 [93] in response to the criticisms by Waskett et al. [22], except that whereas the Erratum states a CI of “0.72–2.98,”his Table 7 states a CI of* “1.72–2.98,”* as also appeared in Van Howe's Reply to critics in 2009 [85]. Table 7 reports an exact OR of 2.26 and the OR in his Erratum was “2.26.” This OR differs from the crude OR of 2.0 and the adjusted OR of 1.6 reported by Cook et al. Incidentally, when transformed to logarithms, 2.26 is the midpoint of 1.72 and 2.98, so “1.72–2.98” may be the CI intended above, hence solving that discrepancy.

The 23 publications listed in his Table 7 vary in quality. For example, one was a high quality multicenter controlled trial in the USA that found higher prevalence of gonorrhea in uncircumcised men after multivariate analyses adjusted for age, race, and site for both cohort data (OR = 1.6; 95% CI 1.0–2.6) and case-control data (OR = 1.3, 95% CI 0.9–1.7) [23]. But Table 7 shows only one set of data for this study, stating an OR of 1.09 (95% CI 0.84–1.44). Cohort studies provide data that is of higher quality than that generated by case-control studies, but Van Howe has ignored these data. His article gives no indication in its Methods section that he would reject poorer quality studies if published alongside higher quality studies, so arguably both results should have been included. He does, however, say,* “When distinct strata of the subjects within a study showed differing outcomes, each strata were considered separately in calculating the summary effect”* (see his page 2, column 1, paragraph 4). If considered as separate strata (which would be only a small expansion of the usual meaning), this would suggest that the approach used should have been as stated in his Methods section.

Listed in Van Howe's Table 7 are observational data [94, 95] showing higher gonorrhea prevalence in uncircumcised men (OR = 1.67; 95% CI 0.50–5.54; and OR = 1.31; 95% CI 0.46–3.75 in each respective study). Curiously though his article neglects to include data from an RCT in Kenya [45] in the meta-analysis, even though he lists this publication in his Table 1 of studies that met the inclusion criteria. Also missing from Table 7 are data from the relevant longitudinal studies in New Zealand [47, 48].

The 27 datasets from the 23 publications in Table 7 are used to derive a random effects summary effect OR of “1.0271” (95% CI “0.86–1.23”). Quite extraordinarily, by plotting the logarithm of the OR against circumcision prevalence in a population (his Figure 2) Van Howe states that,* “As circumcision prevalence approached the extremes, the summary odds ratio in population* [sic]* with a 0% circumcision rate would be estimated at *0.68 (95% CI 0.49–0.96),* while a* [sic]* population with a 100% circumcision rate, the summary odds ratio would be estimated at 1.72 *(95% CI = 1.16–2.55).” This leads him to conclude that,* “the incidence and prevalence of gonorrhea are not affected by circumcision status as much as by the prevalence of circumcision within the community.”* We are not aware of any studies that have compared the protective effect of circumcision by national circumcision prevalence. We agree that there is* “No significant association between the incidence or the prevalence of gonorrhea and circumcision status of males.”*

### 4.6. Genital Herpes/Herpes Simplex Virus Type 2

In Table 10 the article reports finding a random effects summary effect OR of “1.1522” (95% CI “0.95–1.40”) for the meta-analysis of HSV-2. A meta-analysis in 2006 by Weiss et al. found* “reduced risk of HSV-2 infection was of borderline significance (summary *RR= 0.88, 95% CI 0.77 to 1.01)” [4]. Since that time several high quality RCTs have been performed. Given such improvements in the quality of data that have now become available, we would have expected a more definitive conclusion from a meta-analysis in 2013. Disappointingly, in Table 10 (discussed in Van Howe's Section 4.8 on his page 28) we find once again the omission of several relevant studies. This includes two listed in the meta-analysis by Weiss et al. (i.e., [53, 54]), and, more notably, the results from several RCTs. Missing is a trial in Uganda that found an adjusted hazard ratio of 0.72 (95% CI, 0.56–0.92; *P* = .008) [28], and adjusted incidence rate ratio (IRR) of 0.70 (95% CI, 0.55–0.91) [50], a trial in South Africa that reported an IRR of 0.55 (95% CI, 0.32–0.94) [51], and a trial in Kenya that reported an adjusted hazard ratio of 0.94 (95% CI 0.70–1.25) at 2-year followup [49]. After Van Howe's article was published, the Kenyan trial published data for 6 years of followup, revealing a weight adjusted Cox hazard ratio of 0.88 (95% CI 0.77–1.10) [96]. The authors suggested that,* “It is possible that the higher prevalence of infection and greater risk of exposure for younger men in Kisumu overwhelmed any potential moderate or small protective effect of MMC* [medical male circumcision]* against HSV-2”* [96].

While none of these RCT publications appear in Van Howe's Table 10, there was an awareness of two of them: one by Tobian et al. [28] cited in paragraph 2 of his Results section (his reference [82]) and one by Sobngwi-Tambekou et al. [51] (Van Howe's reference [49]) in his Subsection 3.1 which says,* “several reported on redundant study populations.” *We assume that what he meant is that when data in different publications are for the same cohort of men it is sensible to use only one of the studies, so as to avoid giving undue weight to the findings in those men. However, since the meta-analysis did not use* any* data from this Kenyan population in Table 10, Van Howe's statement is fallacious. The article seems unaware that there were two publications by Tobian et al. in 2009 that reported data on HSV-2. One of them [50] is not cited anywhere in Van Howe's article. Tobian et al. concluded that,* “circumcision is efficacious for the prevention of heterosexually acquired HSV-2 infection.”*

Considering the problems outlined above, we suggest that if Van Howe's 2013 meta-analysis had included all of the important data it might have led to a conclusion other than,* “While there is a trend toward higher incidence and prevalence of HSV in intact men, the finding is persistently not statistically significant despite a number of adjustments.” *Moreover, to paraphrase this statement, no matter how hard Van Howe tried to get the result he desired this was not successful. Furthermore, in Section 4.8 he seeks to undermine the results of African studies by saying,* “Using meta-regression, there was a trend *(*P* = .1261)* that odds ratios were higher in African studies.”* Not stated is that this trend falls short of a significant difference.

### 4.7. Genital Discharge Syndrome

Here Van Howe starts off by providing a brief statement in Section 4.1 (page 21) that,* “intact men are more prone to GUD and circumcised men are more prone to GDS,”* concluding,* “Consequently, there is no surprise here.”* It seems odd that such a section should appear, given that the article discusses GDS and GUD separately in Sections 4.2 (Table 4) and 4.6 (Table 8), as we will address separately below.

In the analysis of GDS adjustments are performed based on a funnel plot to justify excluding a study by Warner et al. [97]. This results in a statement that,* “the finding approaches statistical significance (95% *CI*, 0.70–1.03).” *But the analysis showed there was no significant difference. It concludes that, “*the lack of association with intact men and GDS is consistent with what is seen with NSU.”* We therefore refer the reader to the section above that highlights major flaws in the meta-analysis of NGU (formerly termed, as used in Van Howe's article, “NSU”).

### 4.8. Genital Ulcerative Disease (GUD)

Table 8 in Van Howe's article gives a random effects summary effect OR for GUD of* “1.6760” *(95% CI* “1.3926–2.0170”*). In a previous meta-analysis Van Howe stated,* “GUD showed a trend towards being more common in genitally intact men (random-effects summary odds ratio *[OR] = 1.34, 95%* confidence interval *[CI]* = 0.98–1.82). When comparing men with GUD to men with “genital discharge syndrome” (GDS), genitally intact men were more likely to have GUD *(OR = 2.31, 95% CI = 1.70–3.15)*” *[62]. Table 8 lists studies included in the meta-analysis of GUD, and the results are discussed in Subsection 4.6 on page 26 of Van Howe's article.

Table 8 does not list any of the data on GUD from the large high-quality RCTs. Such trial data include a study by Mehta et al. who found circumcision halved GUD [49], and by Gray et al. who found circumcision reduced GUD irrespective of HSV-2 infection [52]. While the paper by Mehta et al. is not cited in Van Howe's article, the publication by Gray et al. is, so there was an awareness of its existence. His reference [53] is amongst twelve that is cited in an unqualified statement in Subsection 3.1 on page 3 that says,* “several reported on redundant study populations.” *The publications by Gray and coworkers that appear in Table 8 are from 2000 to 2004, that is, preceded the reporting of RCT data from this population. While his Table 1 lists Mehta et al. as being an RCT of GUD [98] (Van Howe's reference [172]), instead it studied* Mycoplasma genitalium* (which is likely involved in some NGU) and is not listed in Table 8 of studies of circumcision and GUD. In the trials, anaerobic bacteria were found to be more common in genital ulcers of uncircumcised men [99] and circumcision was found to reduce the total bacterial load and microbiota biodiversity [100]. Circumcision was also found to protect men against* M. genitalium* infection [98].

In Section 3.5.1 Van Howe's article suggests that studies in Africa are the only ones that proved significant for protection against STIs such as GUD. It is well known that various STIs are more prevalent in many sub-Saharan African countries than elsewhere. This makes such countries worthy of study because a high prevalence of an STI should mean a higher power to detect a protective effect of male circumcision against STIs in epidemiological studies. Thus, if there is a difference, then the ability to obtain a statistically significant result should be achieved with a smaller number of subjects. Even though the population effect might be lower, the magnitude of the protective effect of circumcision for individuals per exposure should nevertheless be similar in populations having a lower STI prevalence. There are numerous studies that demonstrate that results for circumcision and protection against HIV and HPV from African studies are applicable to the USA. Warner et al. [97] obtained results for HIV in a Baltimore STI clinic that were similar to the RCTs in Africa. Nielson et al. [101] and Lu et al. [37] obtained results for HPV that were similar to those from the African RCTs referred to above.

Curiously, Van Howe's Subsection 3.5.1 states that the summary effect for GUD was “1.37 (95% CI 1.00–1.85)” for general populations, but omits the OR for high-risk populations, stating only the CI,* “(1.50–2.10)”.*

Given our concerns, Van Howe's meta-analysis of GUD appears unreliable.

### 4.9. Syphilis

In Table 9 his article gives a random effects summary effect OR of “1.3036” (95% CI “1.1103–1.5306”). In Section 4.7 on page 27 where the meta-analysis in Table 9 is discussed, the main* “farrago,”* as elsewhere, is the manner of communication. While referring to different outcomes for association, Van Howe omits to mention with what. Once again we see nonsequiturs such as* “lead-time bias”* and prevalence of syphilis being influenced by circumcision prevalence in the population being studied. Missing from Table 9 is a study by Tabet et al., a study performed in Lima, Peru [55]. This, despite that study being listed in the meta-analysis by Weiss et al. [4] that his article cites. The 2006 meta-analysis by Weiss et al. found that,* “Most syphilis studies reported a substantially reduced risk among circumcised men (summary *RR = 0.67, 95%* confidence interval *(CI)0.54* to *0.83*, although there was significant between study heterogeneity *(*P* = .01)*.”* In the end Van Howe suggests that no conclusion can be made about whether risk of syphilis is higher or lower in uncircumcised men. While his 2013 meta-analysis includes many studies that have been published since the meta-analysis by Weiss et al., its Table 9 does not include any of those data such as those reported in an RCT by Tobian et al., which found no impact of male circumcision on syphilis [28] (Van Howe's reference [82]), the prevalence of which was low in the population studied.

### 4.10. Chancroid

In a meta-analysis of chancroid in 2007 Van Howe concluded,* “There was no difference in the risk for chancroid based on circumcision status *(OR = 0.91, 95% CI = 0.40–2.05)*”* [62]. In contrast, Table 11 of his 2013 article gives a random effects summary effect OR of* “1.33”* (95% CI “0.52–1.33”). Curiously, the CI given for this is “0.52–1.33,” which is clearly incompatible with the OR, in that the upper bound of the CI cannot be the same as the OR. The CI stated would imply an OR of 0.83. His 2007 meta-analysis reported an OR of 0.91 (95% CI, 0.40–2.05).

In his Section 4.9, after performing various adjustments to bring between-study heterogeneity into an acceptable range, Van Howe concludes,* “The data do not support the claim by Weiss et al. that ‘circumcised men are at lower risk of chancroid'.”* Table 11 lists 5 studies, none of which are RCTs. It does not include three of the studies that Weiss and coworkers used for their meta-analysis [4], even though they appear in his Table 1 of studies that met his article's inclusion criteria, namely, Barile et al. [56], which found lower chancroid prevalence in circumcised men (crude risk ratio = 0.04; 95% CI 0.01–0.16), Cameron et al. [57] which also found lower chancroid prevalence in circumcised men (adjusted risk ratio = 0.62; range 0.50–0.76) and Nasio et al. [58] which similarly found lower chancroid prevalence in circumcised men (adjusted risk ratio = 0.66; range 0.35–1.24). While Weiss et al. concluded, “Circumcised men were at lower risk of ch*ancroid in six of seven studies (individual study *RRs: 0.12 to 1.1*),”* they did not carry out a meta-analysis owing to various problems with ascertainment of the outcome and groups being compared in some of the studies. Van Howe's article justifies exclusion of these studies by saying,* “they lacked a direct comparison between intact and circumcised men for a specific diagnosis of chancroid”* and,* “In two of the studies, men with genital ulcers were presumed to have chancroid but never tested for it, while a third study tested the men presumed to have chancroid and found that 31.4% had herpes simplex virus type 2 and only 22.9% had a positive culture for Haemophilus ducreyi.”* Given these severe limitations, Van Howe's meta-analysis of chancroid should be deemed inadequate.

### 4.11. Any Sexually Transmitted Infections

In Section 4.12 on page 31 Van Howe's article argues that his study is novel and unique. Much of this is written in colloquial English using convoluted arguments. The meaning is therefore difficult to grasp. The basic claim appears to be that because the more common STIs are less likely to occur in uncircumcised men, then overall STI prevalence in uncircumcised men is lower, even though some STIs might be higher in uncircumcised men. Of course this depends on the accuracy of the meta-analysis of the most common STIs. Our critique of Van Howe's meta-analysis for each STI, most notably the three types of sexually transmitted urethritis and HPV, shows that his major conclusions are seriously flawed. A more scholarly assessment would be more likely to conclude that overall prevalence of STIs is* higher* in uncircumcised men.

### 4.12. Other Issues Raised

In Van Howe's Discussion (his Section 4.13, “*General Findings*” on page 32, paragraph 2), he argues that, “*If male circumcision has any role (which these analyses also dispute) in reducing the incidence and prevalence of STIs, it should be implemented in easily identifiable high-risk populations*.” His article seems unaware that genital HPV infection is a worldwide epidemic and that there are few low-risk populations. A concern is expressed that, “*A major problem with infant circumcision is the lack of an accurate method of identifying which infants will find themselves in a high-risk population when they become sexually-active*” and underscores the apparent misunderstanding about the ubiquity of HPV infection worldwide and of HIV infection in southern Africa, for example. Crystal ball gazing is required to predict STI epidemiology decades into the future. Some STIs may start to decline in prevalence, which could occur if programs for prevention of HPV by vaccination turn out to be effective. But the decline would be in only those HPV genotypes targeted by the vaccines. The most common HPV genotype identified in the Kenyan RCT was HPV56 (frequency 29%) [33]. This HPV genotype is not covered by the current HPV vaccines. The next most common found in that study was HPV16 (26%), which is one of the two high-risk HPV types that are covered by the HPV vaccines. A study of invasive cervical cancer in Ghana, Nigeria, and South Africa found, however, the most common high-risk HPV types to be those targeted by the vaccines, namely, HPV16 (51.2%) and HPV18 (17.2%) [102]. Another issue is the possibility that an eventual HPV vaccine-mediated reduction in high-risk HPV types 16 and 18 in a population might be accompanied by their replacement with genotypes not covered by the vaccine [103]. Other STIs may increase, which could occur if infant male circumcision is not promoted and implemented as a cheaper, more effective strategy than expensive, time-consuming and challenging programs to circumcise adult males.

Van Howe disputes the clear evidence that circumcision protects against penile cancer (page 32, paragraph 2), stating, “*The lack of a significant association between high-risk HPV infections and circumcision status undermines the argument made by the few who believe that circumcision reduces cancer risk.*” We point out, however, that (i) the evidence of elevated oncogenic HPV genotypes in uncircumcised men, summarized above, document that his claims concerning HPV and penile cancer are incorrect, (ii) his article fails to acknowledge the evidence showing that, besides HPV (which is found in half, not all, of penile cancer cases), the biggest risk factors are phimosis (12-fold increase in risk), balanitis (4-fold), and smegma (3-fold), each of which is either exclusively or largely associated with lack of circumcision, as shown by meta-analyses [5], and (iii) that based on research evidence there is almost universal acceptance in the medical profession that uncircumcised men have the highest prevalence of penile cancer.

Not mentioned is that a history of STIs is associated with elevated risk of prostate cancer and that circumcision prior to sexual debut is associated with lower prostate cancer prevalence [104–107].

Even though HIV was not a topic of his article, Van Howe's Discussion addresses HIV. In so doing, we note fallacious claims about the mechanisms of HIV infection. These deny the extensive epidemiological evidence that has received strong support from research showing the biological basis of HIV infection. Instead his article repeats fallacies that have been resoundingly debunked [67, 108–111]. One of Van Howe's claims is that, “*Langerhans cells are quite efficient in killing HIV cells, which explains the low rate of transmission through sexual contact*” [112]. To quote from the study by De Witte et al. that Van Howe uses as support, “*We observed transmission (to T cells) at a very high viral load, strongly suggesting that Langerin is saturated at high HIV-1 concentrations*”[112]. While Van Howe's statement might be true for low viral loads, a proper understanding of the biological evidence is that exposure to a high load of HIV overwhelms the defense system. Experiments involving foreskin explant cultures have shown the formation of apical viral synapses between cells highly infected with HIV and dendrites of Langerhans cells [113–115]. This process is followed by local HIV budding and HIV capture by Langerhans cells, which takes one hour. Langerhans cells then migrate to the basal layers of the epidermis where they transfer HIV to T cells. In the dermis, T cells infect dendritic cells resulting in systemic infection [113–115]. The inner foreskin is most vulnerable, since HIV-infected cells are unable to penetrate the outer foreskin, which resembles the skin of the rest of the penis [113–115]. Other mechanisms that increase HIV infection risk in uncircumcised men include greater risk of penile tears and increased risk of inflammatory conditions such as balanoposthitis, ulceration, and lesions caused by HSV-2 and HPV.

Van Howe's,* “Missed Studies of Interest”* Subsection 4.14 on pages 32-33 “cherry-picks” several outlier studies that found circumcision had no apparent protective effect against HSV infection in a study of soldiers [84] (his reference [25]) or gonorrhea in sailors [116] (his reference [78]). In pointing out that,* “circumcision status based on country of origin is inexact,”* Van Howe excludes a study that found a higher STI prevalence in immigrant Muslim men than men born in The Netherlands [117] (his reference [117], by coincidence having the same number). While acknowledging that the prevalence of syphilis was lower amongst Jews compared to the rest of the population 80–130 years ago, Van Howe suggests that if circumcision is protective then the prevalence of syphilis should be lower amongst Jewish men than women and then cites studies that found prevalence to be similar in each sex [118, 119] (his references [118, 119]). He suggests that,* “the differences in the rates of lues* [*sic*]* between ethnic groups can be explained by a lack of sexual mixing”* (actually mixing for sexual purposes) [120] (his reference [120], another coincidence in reference numbers). His article deemed these studies unsuitable for inclusion in his meta-analysis and did not list them in his Table 1. While this might be reasonable, Van Howe omits other studies (his references [70, 74–77, 79–81] in Section 3.1) without an adequate explanation, merely saying that while these publications met the inclusion criteria, they,* “did not report their results in a manner to include them in the analyses.”* Some of these should have been included, as we explained above for the RCT data on high-risk HPV and flat penile lesions by Backes et al. [33] (his reference [77]).

Van Howe's Subsection 4.15 entitled* “Methodological Choices”* begins by citing 8 publications by Gisselquist in claiming that in Africa,* “20% or more *[HIV]* infections are not spread through sexual contact,”* ignoring the critiques by experts at WHO, and in academia, that have exposed this claim as being fallacious [67, 108, 121].

Later in this subsection (Van Howe's page 33, column 2, paragraph 4, lines 1-2) his article says,* "Using a control group of men without any STI is problematic*,*”* then in lines 4–6 continues with,* “Some have the mistaken belief that contracting a different STI introduces unidirectional bias”* [22] and in lines 6–12,* “The opposite is likely the case. Excluding men with a different STI is more likely to introduce bias. For example, if, while investigating for association between the prevalence of gonorrhea and circumcision status, all men with syphilis, whether or not they have gonorrhea, are excluded, the measure of association will be biased because intact men presumably have a higher prevalence of syphilis."* Here Van Howe uses an example that differs from the real issue. The issue is whether the control group should include those men who are representative of the general population, or whether it should consist of men suffering from a condition that might itself be associated with circumcision status. But in the example Van Howe uses, men with syphilis are excluded,* “whether or not they have gonorrhea.”* In other words, both cases and controls are modified to exclude cases with another STI.

The next paragraph of the same subsection states (lines 1–7) that, “*Second, using a disease-free control group discards data collected on men who had an STI other than the infection of interest. Those who participate in medical research allow their medical information to be used and their privacy to be violated. Violating a subject's privacy to collect data and then not use the information excludes useful information and is ethically suspect.*” This is perhaps the strangest argument that we have seen in a medical paper. The institutional review board procedures (and the Health Insurance Portability and Accountability Act in the USA) are designed to minimize the risk that research participants' “*privacy is violated.*” Once the primary research paper is published, the ethical obligation to the respective study participants either ends, in the absence of a posttrial phase of service provision, or if follow-up studies are to be undertaken that were not planned initially, then specific permission would be required from institutional review boards [122]. It is simply fallacious to suggest that a third party with no contact with the study participants is somehow ethically obligated to use their data in a subsequent analysis, especially if so doing might introduce bias and thus potentially place future patients at risk.

Van Howe's subsection attacking the use of disease-free control groups continues on page 34, where in paragraph 2 one finds,* “Finally, it provides a method of comparison that is consistent with the other studies included in the meta-analysis."* This is a dubious argument at best. Many studies, if not most, generally use healthy subjects as a control group.

In paragraph 3 on page 34, Subsection 4.15, his article points out in lines 2-3 that, “*Many prefer to use individual patient data in meta-analyses for a variety of reasons,*” and goes on to explain, “*First, not all studies adjust their results for confounding factors.*" This is a very weak argument for using poorer-quality data from studies that do make adjustments. Using the best data wherever they are available provides the highest-quality evidence for any meta-analysis. In lines 4–7, his article continues, “*Second, studies that provided adjusted odds ratios do not consistently adjust for the same factors, so adjusted results from different studies are not comparable*." Again, this is a weak argument. One might as well argue that studies were not drawn from the same populations, so they are not comparable. In any case, Van Howe's article is letting the perfect be the enemy of the good (to paraphrase Voltaire). Even if controlling for the same factors is ideal (and it might not be, if different populations had different kinds of confounding), controlling for any confounding is generally better than not doing so at all.

Continuing on, in lines 7–9, his article states, “*Third, most studies that report adjusted results rarely perform evaluations for collinearity, which can destabilize multivariate models.*" This might perhaps be the strongest argument, but that is not to say very much. Does he mean that the authors failed to evaluate, or failed to report that they did? The two are not the same. Then in lines 13–18, Van Howe says, “*If a study were to adjust for one of these factors, they might find that particular factor is significant, circumcision is significant, or both are significant, when the truth is that circumcision is linked to the other factor and the two variables in a multivariate model are describing the same thing*." But if they are the “*same*” thing then the article is describing perfect collinearity—a situation that is rare in practice. A certain amount of collinearity is expected, and not normally a problem, except where the level of collinearity is high (*R* ^2^ > 0.80) (see [123]). This occurs quite rarely. So in practice, Van Howe is arguing that adjusted ORs should be rejected in case a relatively rare event occurred and the statistician responsible for the analysis was incompetent—a combination that doubtlessly does occur, but must surely be uncommon.

Van Howe goes on to say in lines 18–19, “*Fourth, when adjusted odds ratios are calculated, the uncertainty (variance) of the estimate increases*,” and in lines 29–33, “*Subsequently, a much smaller and less rigorous study that reported only raw data would have more impact on the summary effect than a large nationally representative probability sample using adjusted odds ratios.*" This argument is disingenuous. It is not entirely clear which studies he is referring to. Meta-analyses pay no attention to whether studies are defined as “*less rigorous*” or “*nationally representative.*" Rather, studies are weighted according to the reciprocal of the variance (i.e., very roughly speaking, according to the sample size). Van Howe implies that a study with a large sample size should receive greater weight than should a study with a small sample size. Much depends on the study design and population investigated. It is not uniformly true that a larger sample is better or indeed more certain. There might, for example, be a greater number of confounding factors in the larger population. And if there are confounding factors, then the estimate after adjustment for those factors may indeed be less certain. In such instances it does not seem inappropriate for that study to receive less weight.

Then, in lines 33–36 of this paragraph of Van Howe's Subsection 4.15, he states that, “*Fifth, adjusted odds ratios are open to manipulation using multivariate logistic regression. Consequently, using raw data will diminish the impact of researcher bias and avoid overfitting the data with multivariate analysis.*” Here the article invokes the specter of researcher bias but offers no credible basis as to why the researchers who conducted the particular studies were biased. Logistic regression analyses can be manipulated by unscrupulous researchers, but in the absence of a reason to believe that this is the case, Van Howe's article is effectively welcoming confounding factors on the basis of a remote risk. It is also rather ironic that the article complains about regression analysis being subject to such manipulation in the same paper that it presents metaregressions adjusted for apparently arbitrary data such as the background prevalence of circumcision in the country studied. At a minimum, it would have been advisable to investigate the impact of the decision to use crude ORs by including a secondary analysis using adjusted ORs.

In Subsection 4.16 entitled* “Shortcomings of Meta-Analyses”* he begins by stating,* “Meta-analysis is an inexact tool and best applied to randomized controlled trials.”* Yet, quite extraordinarily, as we have pointed out above for each STI, Van Howe chose to exclude almost all RCT data, thereby undermining the results of his meta-analyses. He states that meta-analysis* “has inherent weaknesses when applied to observational studies,” *yet most of the studies included in his meta-analyses are observational.

We agree with Van Howe's statements that simple inclusion criteria can lead to inclusion of* “studies of less than optimal quality”* and that,* “more exclusive* [does he mean more rigorous?]* criteria can be subject to researcher bias and be manipulated to obtain specific results.”* This indeed sums up our perception of Van Howe's meta-analyses.

His article does point out that one limitation of the systematic review is the “*inability to find all sources of data using any search strategy*”…“*So there may be published and unpublished studies that were not included,*” but this does not excuse Van Howe for listing retrieved studies, then, without justification, failing to include them in his meta-analyses of various STIs.

We further agree that with his statement, “*The trim portion of the “trim and fill” method is handicapped by being based solely on rank, without consideration of study size. Consequently, adjustments for publication bias should be viewed with caution as asymmetry of the funnel plot may be due to factors other than publication bias and, likewise, results generated to correct for the asymmetry may not reflect a correction for publication bias*” [124]. In essence, this confirms our contention that playing with the data in enough ways can lead to almost any result, especially in the hands of pseudoscientists with predetermined agendas.

Finally, the overall criticisms of high quality studies made in Van Howe's Summary (his Section 5) can be dismissed outright as fallacious. While it is not out of the question that, “*high-profile medical journals, such as the New England Journal of Medicine and The Lancet”* might publish articles that,* “contained serious and possibly fatal methodological flaws,” *it is nevertheless unlikely. Several years have now passed since these articles were published and any criticisms have not led to their retraction. It is, moreover, quite specious for Van Howe to make a sweeping final statement that,* “It is clear that any positive impact of circumcision on STIs is not seen in general populations. Consequently, the prevention of STIs cannot be rationally interpreted as a benefit of circumcision, and a policy of circumcision for the general population to prevent STI is not supported by the evidence currently available in the medical literature.” *Detailed review of the data documenting the protective effect of male circumcision against HIV infection led WHO experts to recommend male circumcision as one element of an HIV prevention strategy in high-risk populations. Despite the false results generated by Van Howe's faulty meta-analyses, consideration must be given at the very least to the degree of seriousness of each STI, with high-risk types of HPV for example, being a cause of genital cancer in men and their female sexual partners, and HIV not having been included as one of the STIs his article assessed.

## 5. General Discussion

Our detailed critical examination raises serious doubts about the reliability of Van Howe's meta-analyses that dismiss the protective effects of male circumcision against STIs. Van Howe has previously published a number of meta-analyses on a range of medical conditions that the scientific community judges, based on the scientific evidence, that male circumcision helps prevent. With the possible exception of genital ulcer disease [62], Van Howe's findings from prior meta-analyses have inevitably led to him dismissing the merits of male circumcision as a protective intervention in combating STIs. His previous papers have been criticized by academic experts, who have shown them to contain serious flaws [12, 22, 24, 108, 125]. More scholarly meta-analyses have found that circumcision protects men against a range of STIs, including HSV-2 [4], chancroid [4], syphilis [4], oncogenic types of HPV [5, 24, 87, 91], and HIV [20, 21, 126–129]. Van Howe's meta-analysis of circumcision and HPV [73] led to a critique by Castellsagué et al., which discredited that article's statistical methods, its inclusion criteria and the “sampling bias” issue, concluding that the paper was sufficiently flawed as to warrant retraction from the literature [24]. In that article Van Howe's inclusion criteria were so strict that, of 16 relevant studies, 13 were excluded for questionable reasons, leaving only 3 studies. Two were rejected for failing to sample the penile shaft. Eight were then reincluded, but only after an adjustment had been performed for failing to sample the shaft (using the flawed method that we have highlighted). In the case of sexually transmitted urethritis, Waskett et al. pointed out that some of the source data that appeared in that paper by Van Howe bore little resemblance to the actual source data in the publications the article cited. Adopting NGU as an example, Waskett et al. then proceeded to use the actual source data in a meta-analysis and found that rather than increasing the risk of NGU, this STI was 10% lower in circumcised men, although not reaching statistical significance [22]. In an inadequate reply [85], Van Howe confessed that data from one paper the study drew on* “were improperly extracted”* and apologized, but failed to adequately explain the mismatch in the data from 3 other papers. His present paper has, nevertheless, permitted us to decipher Van Howe's method, which involves manipulation of data to generate different control groups. In the present instance, only a time-consuming check of relevant publications from the 199 he cites in his 42-page article with 17 Tables and 16 Figures would be able to identify misuse of source data once again [10]. Doing so ourselves would take more time than this exercise warrants.

Van Howe's present article completely ignores the previous critiques of both his own work and of sources he cites (e.g., Travis, Gisselquest et al. and Storms). The extensive history of Van Howe's publications is consistent with someone pursuing a deeply ingrained agenda that is opposed to male circumcision and that aims to produce “evidence” to show there is no scientific support for its benefits [9]. The approach he uses differs from the kind of dispassionate, objective, meta-ethical approach that a scientist might normally be expected to adopt in investigation of a research question. As in the present instance, his previous publications seem to be motivated by a desire to create ammunition that fringe groups who are opposed to male circumcision, particularly infant male circumcision, can use in their campaigns [130].

As in the present article, opponents of male circumcision use a nonmedical term* “intact”* instead of “uncircumcised” to describe a penis possessing a foreskin. “Intact” is an emotive term. It implies, incorrectly, that those without a foreskin are lacking something significant, thereby potentially preying on those circumcised men who may be psychologically vulnerable. The term also seems calculated to draw an emotional response from readers, biasing them against circumcision. In contrast, removal of the glans or part thereof would be a significant loss, as would removal of the entire penis. Surgical excision of part, or all, of the penis is often carried out to treat penile cancer, a disease that occurs very much more commonly in uncircumcised men [5, 131]. While removal of the foreskin might technically be regarded as causing the penis to miss an (unimportant [132]) part, operations that remove important parts of the penis render the penis no longer intact. One might refer to children who suffer from ankyloglossia, a condition caused by a short frenum that restricts tongue motion, as having a tongue that is “intact.” Yet surgical intervention for tongue tie is generally regarded as desirable.

## 6. Conclusions

After necessary, detailed scrutiny, we find that Van Howe's arguments and data attempting to discredit the ability of male circumcision to protect against various STIs lack scientific rigour and lead to conclusions that cannot be justified scientifically. They convey an impression of being part of a deliberate, ongoing campaign in support of a deeply ingrained agenda opposed to male circumcision. Van Howe's use of advanced statistical methods not realizing that they lose value when the results are subsequently used in a meta-analysis is reminiscent of the illusion of sophistication apparent in the* “pay no attention to the man behind the curtain”* scene in the* Wizard of Oz*. Conclusions from such analyses are not only erroneous and misleading but could be used to adversely influence health policy and public opinion on a serious medical topic. Articles such as those of Van Howe represent a contamination of the scientific literature and are likely to be held up, falsely, by opponents as “evidence” of the nonefficacy of male circumcision in STI prevention.

Our critique should provide a sound basis for researchers and others to better appreciate the defects that can arise when meta-analyses are not performed well. It is particularly important to realize this because of the importance placed on meta-analyses in development of health policies and for medical decision making.

Since Van Howe's meta-analyses (i) fail to include all relevant studies, especially data from RCTs, (ii) introduce bias into the statistical analyses through use of inappropriate control groups, (iii) involve tampering with the original data, (iv) fail to control for confounders through use of crude odds ratios, (v) use unnecessarily complicated methods with an inadequate explanation of the technical details, thus serving as an impediment to others who might wish to reproduce the analyses, we conclude that his article lacks merit and has generated erroneous conclusions that contradict the scientifically well-established protective effect of male circumcision against a number of common STIs, so making its retraction appropriate. In concluding the debate, we affirm that male circumcision* does* protect against various STIs.

## Figures and Tables

**Table 1:** Examples of important publications not included in the meta-analyses.

Type of STI	Reference to Publication omitted
HPV	[28–33]* [34–39]
Genital warts	[40–44]
*Chlamydia trachomatis *	[45, 46]* [47, 48]
*Neisseria gonorrhea *	[45, 46]* [47, 48]
HSV-2	[28, 49–52]* [53, 54]
GUD	[49, 52]*
Syphilis	[28]* [55]
Chancroid	[56–58]

*Randomized controlled trials.

**Table 2:** Sampling sites as stated in various studies that tested for HPV.

Publication	Site(s)
Aynaud et al., 1999 [41]	Urethra
Weaver et al. 2004 [59]	Urine, glans, shaft, scrotum, foreskin
Flores et al., 2008 [60]	Combined glans/sulcus, combined shaft/foreskin, scrotum,
	Perineum, anus, urethra, semen
Auvert et al., 2009 [31]	Urethra
Lu et al., 2009 [37]	Combined glans/sulcus, shaft, scrotum
Tobian et al., 2009 [28]	Preputial cavity (uncircumcised men); coronal sulcus (circumcised men)
Gray et al., 2010 [32]	Combined glans/sulcus
Hernandez et al., 2010 [61]	Combined glans/sulcus, shaft, scrotum, inner foreskin
Tobian et al., 2011 [29]	Sulcus, shaft
VanBuskirk et al. 2011 [39]	Urine, glans/corona, shaft/scrotum
Backes et al., 2012 [33]	Combined shaft/outer foreskin, combined glans/corona/inner foreskin
Tobian et al., 2012 [30]	Combined glans/sulcus

**Table 3:** Comparison of OR in the 2013 and 2007 meta-analyses with the actual figures in four of the studies cited in each. See Section 2.2 for how use of crude ORs and Section 3.5 for how use of different control groups account for some of the discrepancies.

Condition	Study*	OR (95% CI) for association with lack of circumcision		
		Actual^†^	Van Howe 2007 [62]	Van Howe 2013^§^ [10]
NSU	Cook	1.0 (0.8–1.3)	0.40 (0.26–0.62)	0.89 (0.73–1.10)
	Dave	0.85 (0.54–1.35)	0.88 (0.61–1.25)	0.88 (0.61–1.25)
	Laumann	0.72 (0.24–2.33)	0.77 (0.45–1.34)	0.77 (0.45–1.34)
	Parker	1.08 (0.81–1.44)	0.64 (0.50–0.82)	0.64 (0.50–0.82)

Chlamydia	Cook	1.0 (0.7–1.6)	0.90 (0.60–1.36)	0.95 (0.65–1.40)
	Dave	1.23 (0.62–2.44)	1.22 (0.66–2.26)	1.22 (0.66–2.26)
	Laumann	Not stated^¶^	0.02 (0.00–0.28)	0.02 (0.00–0.28)
	Parker	1.12 (0.71–1.75)	1.02 (0.65–1.59)	1.02 (0.65–1.59)

Gonorrhea	Cook	1.6 (1.2–2.2)	2.74 (1.98–3.80)	2.26 (1.72–2.98)
	Dave	0.76 (0.39–1.49)	1.22 (0.66–2.26)	0.71 (0.40–1.27)
	Laumann			
	1–4 partners	0.45 (0.05–4.0)	0.92 (0.39–2.21)	0.92 (0.39–2.21)
	5–20 partners	1.27 (0.55–2.63)	1.39 (0.95–2.04)	1.39 (0.95–2.04)
	21+ partners	0.31 (0.09–1.04)	0.78 (0.49–1.25)	0.78 (0.49–1.25)
	Parker	2.29 (1.48–3.56)	1.61 (1.06–2.44)	1.61 (1.06–2.44)

*Shown is first author of each study: Cook et al. [63], Dave et al. [64], Laumann et al. [65], Parker et al. [66].

^†^Shown is adjusted OR provided in Cook et al. [63], Laumann et al. [65], and Parker et al. [66]. Since Dave et al. [64] only provided an OR we show the latter instead. Since Dave et al. [64] and Laumann et al. [65] expressed this as association with circumcision rather than lack of circumcision the value shown as been inverted for consistency across all studies.

^§^The 2013 meta-analysis expresses ORs as association with lack of circumcision.

^¶^Laumann et al. [65] do not provide an OR for Chlamydia. See text for the method we suggest the 2013 article used to derive the values presented.
